# One, two and three-dimensional ultrasound measurements of carotid atherosclerosis before and after cardiac rehabilitation: preliminary results of a randomized controlled trial

**DOI:** 10.1186/1476-7120-11-39

**Published:** 2013-11-06

**Authors:** Tamas J Lindenmaier, Daniel N Buchanan, Damien Pike, Tim Hartley, Robert D Reid, J David Spence, Richard Chan, Michael Sharma, Peter L Prior, Neville Suskin, Grace Parraga

**Affiliations:** 1 Imaging Research Laboratories, Robarts Research Institute, 1151 Richmond St, London, ON N6A 5B7, Canada; 2 Department of Medical Biophysics, The University of Western Ontario, London, Canada; 3 Division of Cardiology, Department of Medicine, The University of Western Ontario, London, Canada; 4 Division of Prevention and Rehabilitation, University of Ottawa Heart Institute, Ottawa, Canada; 5 Department of Clinical Neurological Sciences, The University of Western Ontario, London, Canada; 6 Cardiac Rehabilitation and Secondary Prevention, St. Joseph’s Hospital, London, Canada

**Keywords:** Carotid ultrasound, Comprehensive cardiac rehabilitation, Transient ischemic attack, Ischemic stroke, Three-dimensional ultrasound, Intima-media thickness, Total plaque area, Total plaque volume, Vessel wall volume

## Abstract

**Background:**

It is still not known how patients who are post-transient ischemic attack (TIA) or post-stroke might benefit from prospectively planned comprehensive cardiac rehabilitation (CCR). In this pilot evaluation of a larger ongoing randomized-controlled-trial, we evaluated ultrasound (US) measurements of carotid atherosclerosis in subjects following TIA or mild non-disabling stroke and their relationship with risk factors before and after 6-months of CCR.

**Methods:**

Carotid ultrasound (US) measurements of one-dimensional intima-media-thickness (IMT), two-dimensional total-plaque-area (TPA), three-dimensional total-plaque-volume (TPV) and vessel-wall-volume (VWV) were acquired before and after 6-months CCR for 39 subjects who had previously experienced a TIA and provided written informed consent to participate in this randomized controlled trial. We maintained blinding for this ongoing study by representing treatment and control groups as A or B, although we did not identify which of A or B was treatment or control. Carotid IMT, TPA, TPV and VWV were measured before and after CCR as were changes in body mass index (BMI), total cholesterol (TC), high-density lipoprotein (HDL), low-density lipoprotein (LDL), triglycerides (TG), systolic blood pressure (SBP) and diastolic blood pressure (DBP).

**Results:**

There were no significant differences in US measurements or risk factors between groups A and B. There was no significant change in carotid ultrasound measurements for group A (IMT, p = .728; TPA, p = .629; TPV, p = .674; VWV, p = .507) or B (IMT, p = .054; TPA, p = .567; TPV, p = .773; VWV, p = .431) at the end of CCR. There were significant but weak-to-moderate correlations between IMT and VWV (r = 0.25, p = .01), IMT and TPV (r = 0.21, p = .01), TPV and TPA (r = 0.60, p < .0001) and VWV and TPV (r = 0.22, p = .02). Subjects with improved TC/HDL ratios showed improved carotid VWV although, this was not statistically significant.

**Conclusion:**

In this preliminary evaluation, there were no significant differences in carotid US measurements in the control or CCR group; a larger sample size and/or longer duration is required to detect significant changes in US or other risk factor measurements.

## Background

Cerebrovascular diseases are one of the leading causes of death worldwide and are estimated to account for 10% of all deaths [1]. In particular, patients who experience a transient ischemic attack (TIA) are at high risk of stroke and have increased mortality rates [2]. Nevertheless, evidence from clinical trials indicates that risks of a subsequent stroke can be reduced [2] and it is clear that earlier diagnosis and intervention are critical for lowering both the immediate and ongoing risk.

Carotid ultrasound (US) provides a direct and cost-effective way to quantify surrogate markers of carotid atherosclerosis [3-5]. A number of US measurements have been developed to monitor the progression and regression of atherosclerosis, including one-dimensional (1D) intima-media thickness (IMT), two-dimensional (2D) total plaque area (TPA) [5], as well as three-dimensional ultrasound (3DUS) total plaque volume (TPV) [6-10] and vessel wall volume (VWV) [7,8,11-15]. Carotid artery IMT has been shown in meta-analyses to weakly predict ischemic stroke events, while progression of IMT does not [16,17]. Measurement of carotid plaque burden using 3DUS carotid TPA or TPV has also been investigated [18,19] and recently [20] progression of TPV was shown to be a significant predictor of outcomes such as TIA, stroke, myocardial infarction and death, whereas progression of IMT was not.

A feasibility study of the effect of comprehensive cardiac rehabilitation (CCR) on patients with a recent TIA or mild non-disabling stroke (MNDS) [21] showed significant improvements after 6 months CCR in total cholesterol (TC), total cholesterol/low-density lipoprotein (TC/LDL), triglycerides (TG), waist circumference (WC), body mass index (BMI) and body weight. There was also a trend towards improved low-density lipoprotein (LDL), systolic blood pressure (SBP) and diastolic blood pressure (DBP) [21]. Unanswered research questions stemming from this important pilot study remain*: 1) Does carotid atherosclerosis change during 6 month CCR?, 2) Are potential changes in carotid plaque over the course of CCR correlated with changes in serum biomarkers?, and, 3) Do baseline carotid ultrasound measurements predict changes in US and other risk factors after 6–months CCR?*

Our objective was to investigate carotid ultrasound measurements of atherosclerosis in one, two and three-dimensions in a small group of subjects, before and after 6 months of CCR, to evaluate changes over time and to explore potential correlations between stroke risk factors and carotid US measurements. In this proof –of-concept study, our overarching hypothesis was that CCR intervention slowed atherosclerosis progression. Here we directly tested the hypothesis that after 6-months CCR, there would be significantly improved US measurements of carotid atherosclerosis in subjects with improved cardiovascular risk, measured using their serum lipid profile.

## Methods

### Inclusion and exclusion criteria

All thirty-nine subjects provided written informed consent to 3DUS as part of an ongoing, two-site (London, n = 60 and Ottawa, n = 37) randomized controlled trial of 6-months of CCR following TIA. The study protocol was approved by Health Sciences Research Ethics Board, The University of Western Ontario London Canada. Briefly, subjects with a documented TIA or a MNDS within the previous 3 months were recruited. Subjects were at least 20 years of age, spoke and understood English, and also had one or more of the following risk factors: hypertension, diabetes mellitus, dyslipidemia, ischemic heart disease or cigarette smoking within the past year. In addition, subjects who were unable to perform CCR exercises or had already participated in CCR were not enrolled. Subjects were excluded from analyses if they did not have carotid US images with sufficient image quality to perform IMT, TPA, TPV or VWV measurements.

### Intervention

A detailed description of the CCR intervention was previously reported [21]. Briefly, a two-hour orientation session led by a nurse case manager was undertaken for subjects who provided consent [21]. The orientation session provided risk factor education, screening with the Hospital Anxiety & Depression Scale [22] and a quality of life evaluation using the SF-12 Health Survey [23]. Subjects were also advised on smoke cessation and after a 12 hour overnight fast, tested for lipids and fasting blood glucose [21]. Prior to CCR, medical and risk factor assessment was performed [21] and subjects were provided a choice of CCR intervention -on-site exercise twice weekly for 50 sessions with complementary in-home exercise at least 2 days a week, or in-home exercise at least 4 days a week with monthly follow-up by telephone or on-site [21].

### Data acquisition

Carotid artery 3DUS was acquired with a high resolution B-mode ultrasound system (ATL HDI 5000; Philips, Bothel, WA, USA) and an 8.5 MHz transducer (Philips) at the Stroke Prevention & Atherosclerosis Research Centre in London, Canada as previously described [24]. To briefly summarize, the US transducer was manually placed on the neck and then moved along a 5 cm section of the neck mechanically, acquiring a series of 2D images that were reconstructed into a 3D volume. To obtain high quality images, the sonographer optimized the imaging parameters of the system for each subject independently. At baseline (BL) and follow-up (FU), 3DUS volumes of the left and right carotid artery were acquired and archived for measurements made offline.

### Intima media thickness

IMT measurements (ICC = 0.95) [7] were performed by a single observer as previously described [4]. A single observer who was blinded to subject identity and time-point generated a single 2D longitudinal view of the carotid artery from the 3DUS image. The resultant 2D image was segmented and evaluated using Prowin 24.0 (Medical Technologies International Inc, Palm Desert, CA, USA) previously developed and validated [25]. IMT was measured five times for each carotid side and the mean measurements were averaged to provide a value for the left and right carotid artery for each subject.

### 3DUS Total plaque area

TPA measurements were generated in real-time by an experienced sonographer (ICC = 0.94) [5] at the time of the 3DUS image acquisition using the trackball of the ultrasound system as previously described [5]. Briefly, the transducer was positioned to obtain a longitudinal view of the plaque at its greatest footprint in the image volume. The outer boundaries of each individual plaque was segmented manually in that plane and all plaque areas were summed to generate a TPA measurement for the left and right carotid artery for each subject. The sonographer was blinded to treatment status and previous TPA measurements at the follow-up scanning session.

### 3DUS Total plaque volume

A single observer (the same for IMT, TPV and VWV) also generated TPV (ICC = 0.85) [8] for BL and FU images. Randomized and blinded BL and FU images were simultaneously displayed on adjacent monitors to ensure identification of the same plaque in images at both time-points. For the generation of TPV measurements, custom-built software tools previously described [26] were used for evaluation of carotid plaque identified within 10 mm distal to the bifurcation in the common carotid artery (CCA) and 5 mm proximal to the BF in the internal carotid artery (ICA). TPV for each individual was calculated by summing the volume of individual plaques within the left and right carotid arteries.

### 3DUS Vessel wall volume

The same single observer evaluated blinded BL and FU images for 3DUS VWV using a semi-automated segmentation method (ICC = 0.95) [8], previously described [15]. Briefly, the media-adventitia boundary (MAB) and the lumen-intima boundary (LIB) were manually segmented in 11 slices of the CCA and 6 slices of the ICA with an inter-slice distance of 1 mm [24]. Using the inter-slice distance and the areas enclosed by the MAB and LIB in each slice, the volumes between slices were calculated. Measurements of the left and right sides were summed together to obtain a VWV for each individual.

### Statistical analysis

Since our analysis is part of a larger ongoing randomized control trial, the CCR and control subject data were kept blinded and named as group A or B in order to eliminate the potential for bias in future analyses. Statistical analysis was performed using IBM SPSS Statistics for Windows v20 (IBM Corp, Armonk, NY, USA 2011) and GraphPad Prism for Windows, v6.02 (GraphPad Software, La Jolla, CA, USA). The Shapiro-Wilk test was used to assess for normality of samples. Student’s parametric *t*-tests were performed to assess baseline differences between groups A and B, to detect any differences between BL and FU ultrasound measurements, and to detect any differences in US measurements between groups A and B after the treatment period. In the case of non-parametric samples, the Mann–Whitney test was used to assess differences. Changes in plaque burden over the course of CCR, regardless of group assignment, were determined for subjects with an improved lipid profile using Student’s *t*-test, and Spearman correlations between carotid plaque burden and risk factors were determined. Results were considered significant when the probability of making a type I error was less than 5% (*p* < .05). The Holm-Bonferroni correction was used for multiple comparisons.

## Results

### Subject characteristics

Table 1 provides a summary of the baseline study characteristics for all 39 enrolled subjects. In Table 2, a subject listing for all 39 subjects is provided with BL and FU ultrasound and demographic measurements. Mean age was 65 ± 10 years, and there were no significant differences between group A and group B. The CCR and control therapy period was 223 (±66) days.

**Table 1:** Study subject baseline characteristics

	**All evaluated**		**Evaluated by group**				
			**Group A**		**Group B**		***p***
	***n***	**Mean (SD)**	***n***	**Mean (SD)**	***n***	**Mean (SD)**	
Age year (±SD)	39	65 (10)	19	64 (10)	20	66 (11)	1.00
BMI kg/m^2^ (±SD)	39	28 (4)	19	29 (4)	20	26 (4)	0.07
TC mmol/L (±SD)	39	4.24 (1.23)	19	4.10 (1.13)	20	4.37 (1.33)	1.00
HDL mmol/L (±SD)	39	1.34 (0.46)	19	1.29 (0.35)	20	1.38 (0.55)	1.00
LDL mmol/L (±SD)	39	2.28 (0.97)	19	2.21 (0.95)	20	2.35 (1.00)	1.00
TG mmol/L (±SD)	39	1.35 (0.67)	19	1.29 (0.52)	20	1.41 (0.79)	1.00
SBP mmHg (±SD)	39	125 (13)	19	124 (14)	20	125 (11)	0.99
DBP mmHg (±SD)	39	74 (10)	19	77 (10)	20	71 (10)	0.41
IMT mm (±SD)	36	0.762 (0.133)	17	0.785 (0.121)	19	0.742 (0.142)	1.00
TPA cm^2^ (±SD)	36	1.12 (1.25)	19	1.05 (1.64)	17	1.19 (0.64)	1.00
TPV mm^3^ (±SD)	32	100 (101)	14	55 (60)	18	137 (115)	0.20
VWV mm^3^ (±SD)	29	929 (193)	13	972 (234)	16	894 (152)	1.00

BMI = body mass index; TC = total cholesterol; HDL = high density lipoprotein; LD = low density lipoprotein; TG = triglycerides; SBP = systolic blood pressure; DBP = diastolic blood pressure; IMT = intima-media thickness; TPA = total plaque area; TPV = total plaque volume; VWV = vessel wall volume.

**Table 2:** Subject summary

**Sub.num.**	**Group**	**Sex**	**Ageyrs**	**BMI BLkg/m**^**2**^	**TC BL mmol/L**	**TG BL mmol/L**	**HDL BL mmol/L**	**LDL BLmmol/L**	**SBP BL mmHg**	**DBP BL mmHg**	**IMT BLmm**	**IMT FUmm**	**TPA BLcm**^**2**^	**TPA FUcm**^**2**^	**TPV BLmm**^**3**^	**TPV FUmm**^**3**^	**VWV BLmm**^**3**^	**VWV FUmm**^**3**^
1	A	M	54	32.5	4.8	0.71	1.5	2.98	124	80	0.670	0.644	0.00	0.00	0	0	1040	1006
2	B	M	59	29.1	4.0	2.50	0.8	2.12	122	76	0.655	0.62	0.92	0.73	106	132	846	725
3	B	F	45	24.2	4.9	0.70	2.2	2.40	146	82	0.658	0.728	0.03	0.03	23	0	591	613
4	A	F	61	24.9	3.6	0.58	2.0	1.37	112	80	0.641	0.705	0.15	0.10	26	25	776	789
5	B	F	60	33.8	3.9	1.17	0.8	2.51	118	62	0.526	0.622	1.37	n/a	135	130	906	912
6	A	M	56	30.6	3.9	1.75	1.0	2.03	128	78	0.762	0.679	1.41	1.75	n/a	n/a	n/a	n/a
7	A	F	63	32.7	6.5	2.18	1.4	4.17	130	80	n/a	n/a	0.29	0.26	n/a	n/a	n/a	n/a
8	B	F	41	19.8	6.4	0.80	2.4	3.59	110	50	0.514	0.536	0.29	0.33	0	0	709	670
9	B	M	61	27.9	6.1	1.98	1.5	3.72	140	80	n/a	n/a	1.98	1.97	n/a	n/a	n/a	n/a
10	B	M	77	21.8	3.5	0.59	1.4	1.79	139	58	0.953	0.932	1.24	1.68	479	472	1007	1055
11	A	M	68	29.5	4.0	1.88	1.0	2.11	150	76	0.969	0.86	0.52	0.61	100	83	1534	1548
12	B	F	82	27.4	4.7	1.44	1.8	2.24	120	70	0.792	0.847	1.22	1.30	132	150	786	936
13	A	M	71	27.6	5.0	0.99	2.2	2.43	150	86	0.902	0.855	1.52	1.47	115	82	985	1030
14	B	F	69	27.4	5.2	1.83	1.4	2.94	122	56	0.740	0.894	n/a	1.61	137	156	1206	1215
15	B	F	73	22.3	4.5	1.39	1.6	2.26	136	80	0.606	0.732	0.53	0.39	24	17	n/a	n/a
16	A	M	51	24.3	4.7	1.09	1.3	2.93	118	80	0.657	0.651	0.11	0.09	0	0	976	916
17	A	F	75	33.6	3.1	1.82	1.1	1.18	122	66	0.804	0.666	0.13	0.38	0	0	1112	1089
18	B	M	64	29.7	6.0	3.84	1.3	2.93	140	68	0.720	0.956	1.58	n/a	154	122	1112	1023
19	B	M	64	29.0	5.4	2.25	0.8	3.55	118	76	0.829	0.669	0.94	0.98	9	14	876	924
20	A	F	66	34.3	4.6	0.99	1.3	2.79	110	75	0.865	0.744	0.32	0.35	n/a	n/a	n/a	n/a
21	A	M	56	32.0	4.2	0.81	1.3	2.51	110	80	0.655	0.671	0.29	0.30	107	92	1172	1187
22	B	M	68	28.4	5.2	1.06	0.9	3.83	124	82	0.669	0.757	1.53	1.84	189	208	868	807
23	A	M	68	28.1	4.9	2.05	0.9	3.01	140	90	0.736	0.851	0.61	0.51	0	0	815	848
24	A	M	75	28.3	2.7	1.20	0.9	1.29	115	61	0.834	0.926	1.91	2.41	n/a	n/a	n/a	n/a
25	B	F	77	20.5	4.8	0.81	2.4	2.02	118	60	0.662	0.636	1.76	1.39	192	185	1006	1069
26	A	F	82	31.5	6.0	1.61	1.5	3.77	142	84	n/a	n/a	0.38	0.31	0	0	1074	1104
27	A	M	52	26.0	4.5	1.61	1.7	2.04	130	90	0.740	0.81	0.92	0.78	29	30	n/a	n/a
28	A	M	62	24.1	2.0	1.32	1.0	0.30	104	50	0.942	0.925	7.37	6.60	n/a	n/a	n/a	n/a
29	B	M	71	23.9	2.4	0.60	1.0	1.10	130	80	0.949	1.107	2.24	2.18	n/a	n/a	n/a	n/a
30	A	F	57	34.0	3.1	0.64	1.1	1.69	120	80	0.999	0.904	0.98	1.11	57	86	688	737
31	B	M	61	27.4	1.9	0.72	0.7	0.82	110	80	0.718	0.822	1.06	1.00	217	254	834	889
32	A	M	80	21.9	2.7	0.76	1.3	1.07	100	70	0.661	0.687	1.64	1.47	176	180	702	629
33	B	F	80	20.5	2.6	0.88	1.3	0.85	110	60	1.044	0.914	0.79	0.48	17	26	774	814
34	B	M	77	26.4	3.0	1.20	1.4	0.97	110	70	0.902	0.823	1.38	0.91	215	205	917	953
35	B	M	59	24.8	2.7	1.40	0.8	1.28	128	76	0.770	0.829	1.04	0.65	97	81	1015	948
36	A	M	61	28.9	3.8	0.79	1.3	2.14	130	80	0.847	0.973	1.16	1.71	132	135	1005	829
37	A	F	50	32.1	3.9	1.66	1.0	2.18	130	80	0.658	0.669	0.27	0.37	27	34	752	765
38	B	M	65	30.2	5.1	1.40	2.1	2.36	124	78	0.685	0.823	0.89	0.91	103	107	851	996
39	B	M	63	20.8	5.4	1.62	1.0	3.64	126	66	0.712	0.778	2.36	2.79	238	228	n/a	n/a

### Carotid ultrasound measurements

**Figure 1:**
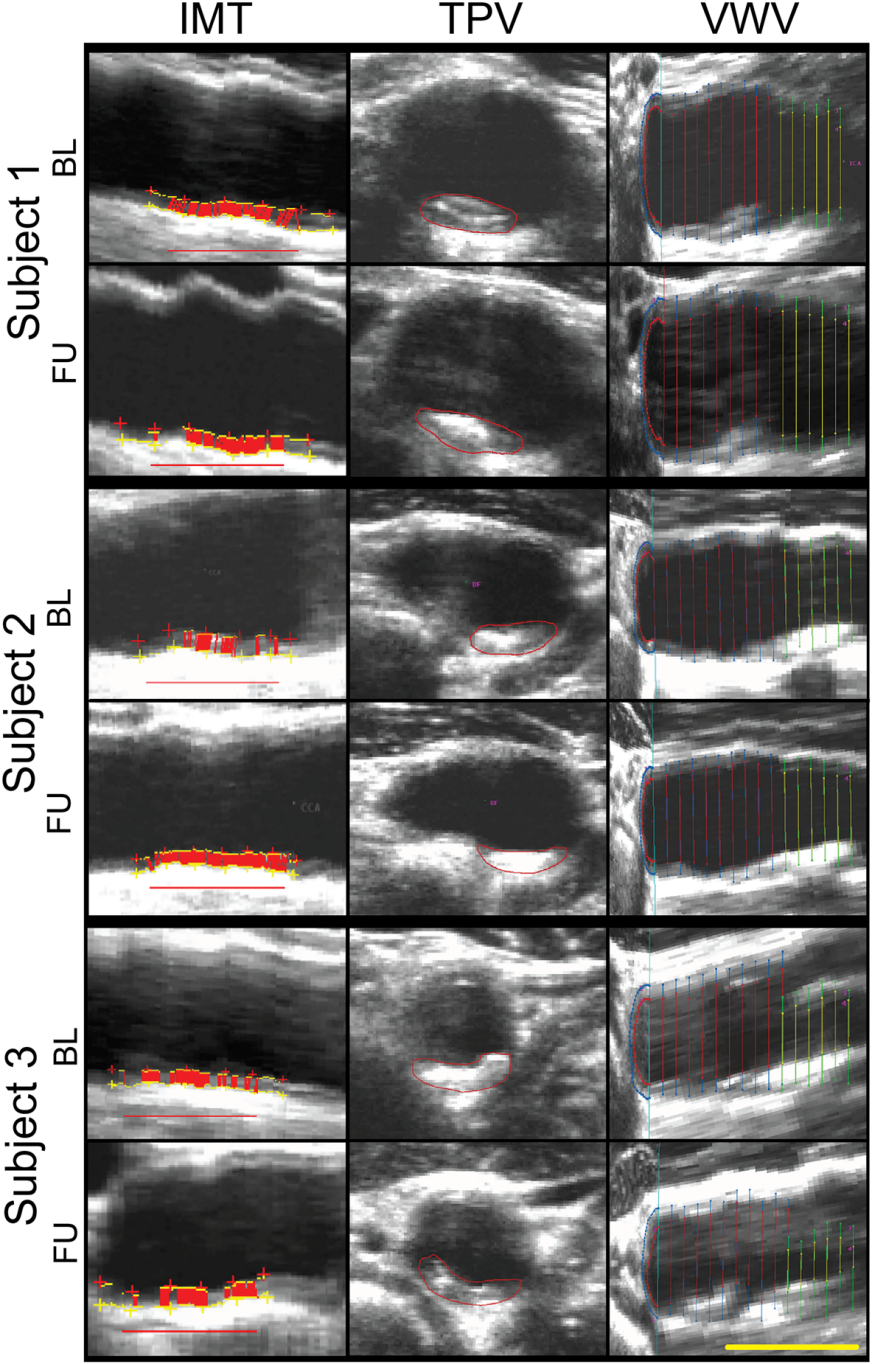
**Carotid Atherosclerosis Measurements for Three Representative Subjects.** Baseline and 6 month follow-up for intima media thickness (left column), total plaque volume (middle column), and vessel wall volume (right column). Scalar bar = 1 cm. Subject 1 is a 68-year-old male from Group A; R carotid artery baseline IMT = 1.044 mm, TPV = 100 mm^3^, VWV = 710 mm^3^ and follow-up IMT = 0.862 mm, TPV = 80 mm^3^, VWV = 720 mm^3^. Subject 2 is a 77-year-old male from Group B with R carotid artery baseline IMT = 0.895 mm, TPV = 70 mm^3^, VWV = 390 mm^3^ and follow-up IMT = 0.790 mm, TPV = 70 mm^3^, VWV = 420 mm^3^. Subject 3, from Group B is a 61 year old male; R Carotid artery IMT = 0.744 mm, TPV = 170 mm^3^, and VWV = 430 mm^3^ at baseline and IMT = 0.882 mm, TPV = 200 mm^3^ and VWV = 470 mm^3^ at follow-up.

Figure 1 shows carotid artery IMT, TPV, and VWV images and measurements for three representative subjects at BL and after six months FU. Subject 1 is a 68 year old male from group A with decreased right carotid artery IMT (0.182 mm) and TPV (20 mm^3^), but a slightly increased VWV (10 mm^3^). Subject 2, is a 77 year old male from group B with minimal changes in TPV and VWV of the right carotid over the CCR period. In contrast Subject 3, a 61 year old male from group B, showed an increase in right carotid IMT (0.138 mm), TPV (30 mm^3^) and VWV (40 mm^3^).

**Figure 2:**
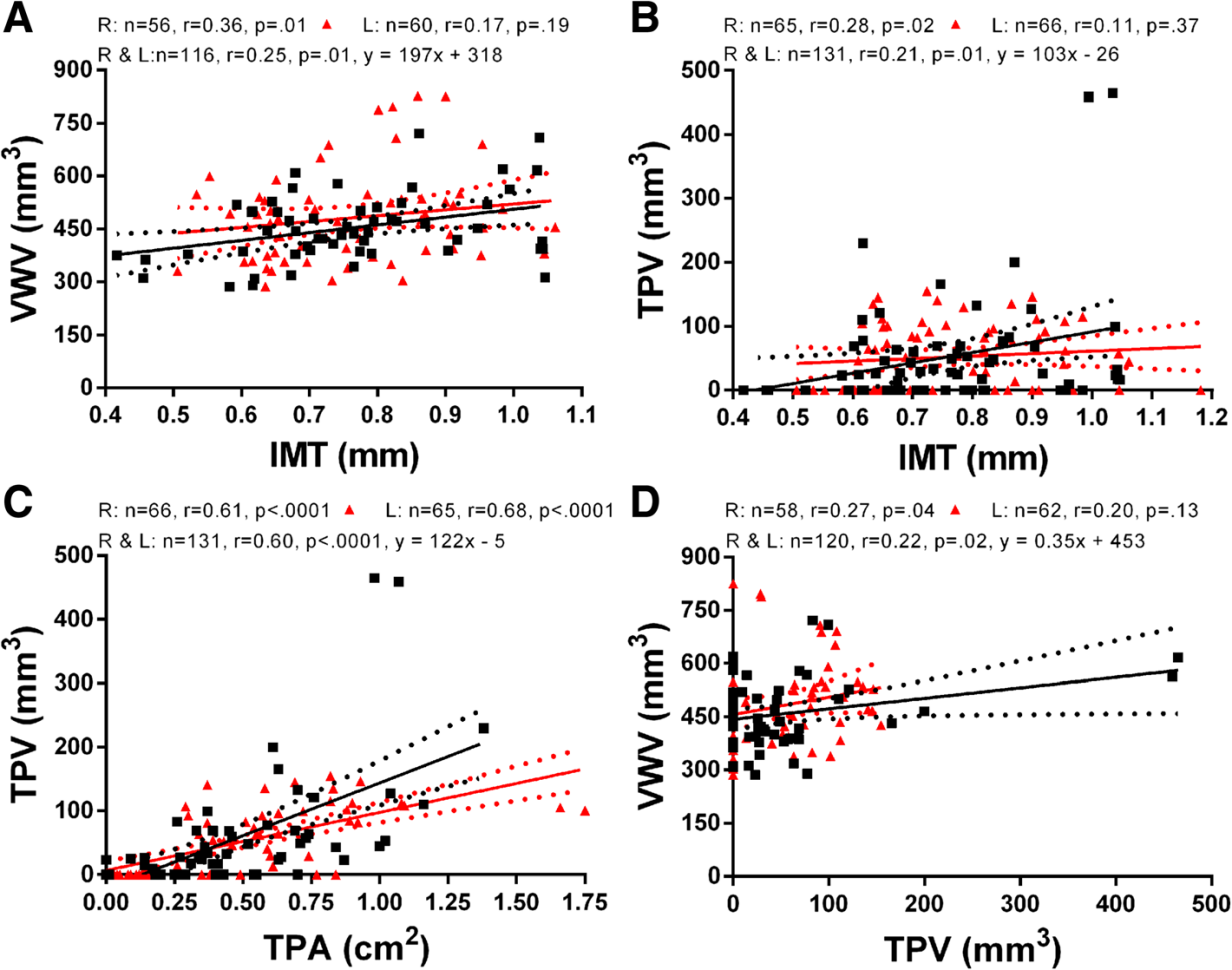
**Relationships for Carotid Ultrasound Measurements.** Panels **A-D** show relationships for ultrasound (US) measurements in 39 subjects, including intima-media thickness (IMT), total plaque area (TPA), total plaque volume (TPV) and vessel wall volume (VWV) for Left and Right carotids at baseline and follow-up. Panel **A** and **B** show the association of IMT with VWV and TPV. Panel **C** shows relationship of TPV with TPA and panel **D** shows the association between VWV and TPV.

Table 3 summarizes the BL and FU carotid ultrasound measurements for groups A and B; there were no significant differences for IMT, TPA, TPV and VWV after CCR. In Figure 2, correlations are shown for ultrasound measurements for all subjects. Modest but significant correlations were observed for VWV and IMT (r = 0.25, p = .01), TPV and IMT (r = 0.21, p = .01), TPV and TPA, (r = 0.60, p < .0001) and finally for VWV and TPV (r = 0.22, p = .02) for all left and right carotid sides. There were similar findings for right (R) (VWV and IMT, r = 0.36, p = .01; TPV and IMT, r = 0.28, p = .02; TPV and TPA, r = 0.61, p < .0001; VWV and TPV, r = 0.27, p = .04), and left (L) (TPA and TPV; r = 0.68, p < .0001) carotid artery measurements. Significant correlations were also observed for all males only between TPV and TPA (r = 0.56, p < .0001). For females only, significant correlations were also observed between TPV and TPA (r = 0.83, p < .0001) and between VWV and TPV(r = 0.47, p = .001).

**Table 3:** Baseline and follow-up carotid ultrasound measurements

**Measurement (±SD)**	**Group A**					**Group B**				
	***n***	**BL**	**FU**	**Total diff.**	***p***	***n***	**BL**	**FU**	**Total diff.**	***p***
IMT (mm)	17	0.785 (0.121)	0.778 (0.115)	-0.007 (0.083)	0.728	19	0.742 (0.142)	0.791 (0.140)	0.048 (0.103)	0.054
TPA (cm^2^)	19	1.05 (1.64)	1.08 (1.50)	0.03 (0.28)	0.629	17	1.19 (0.64)	1.15 (0.74)	-0.04 (0.27)	0.567
TPV (mm^3^)	14	55 (60)	53 (57)	-2 (14)	0.674	18	137 (115)	138 (116)	1 (18)	0.773
VWV (mm^3^)	13	972 (234)	960 (241)	-12 (62)	0.507	16	894 (152)	909 (156)	15 (76)	0.431

TPA = total plaque area; IMT = intima media thickness; TPV = total plaque volume; VWV = vessel wall volume; BL = baseline; FU = follow-up.

Total IMT is calculated by taking the mean between the left (L) and right (R) sides. Total TPA, TPV and VWV is the sum of the L and R sides.

### Risk factors and carotid US measurements

Table 4 provides a summary of baseline carotid ultrasound measurements correlations with their change and the change in risk factors after completion of the 6-month treatment period. There were no significant correlations between any of the US measurements and change in risk factors. As shown in Table 5, there were no significant correlations for the change in US measurements and the change in risk factors. Furthermore, as shown in Table 6 there were no significant differences for the change in US measurements over time, between subjects with improved and unchanged TC/HDL ratios.

**Table 4:** Relationships for baseline US measurements and risk factors

**Risk factors**		**IMT (mm)**			**TPA (cm**^**2**^**)**			**TPV (mm**^**3**^**)**			**VWV (mm**^**3**^**)**	
	***n***	***r***	***p***	***n***	***r***	***p***	***n***	***r***	***p***	***n***	***r***	***p***
	**Group A**											
∆ IMT (mm)	17	-0.44	0.88	17	0.23	1.00	13	0.17	1.00	12	-0.30	1.00
∆ TPA (cm^2^)	17	0.24	1.00	19	-0.10	1.00	14	0.06	0.85	13	0.24	1.00
∆ TPV (mm^3^)	13	0.08	0.79	14	0.22	1.00	14	0.07	1.00	13	-0.64	0.15
∆ VWV (mm^3^)	12	0.34	1.00	13	0.25	1.00	13	-0.10	1.00	13	-0.04	0.91
∆ BMI (kg/m^2^)	16	-0.15	1.00	18	0.35	1.00	13	0.27	1.00	12	-0.21	1.00
∆ TC (mmol/L)	16	0.25	1.00	18	0.31	1.00	13	0.09	1.00	12	-0.45	1.00
∆ HDL (mmol/L)	16	0.20	1.00	18	0.39	1.00	13	0.26	1.00	12	-0.34	1.00
∆ LDL (mmol/L	16	0.11	1.00	18	0.06	0.82	13	-0.15	1.00	12	-0.29	1.00
∆ TG (mmol/L)	16	0.30	1.00	18	0.46	0.58	13	0.40	1.00	12	-0.21	1.00
∆ SBP (mmHg)	16	-0.10	1.00	18	-0.10	1.00	13	-0.14	1.00	12	-0.12	1.00
∆ DBP (mmHg)	16	0.13	1.00	18	-0.06	1.00	13	-0.32	1.00	12	0.63	0.30
	**Group B**											
∆ IMT (mm)	19	-0.21	1.00	16	0.06	1.00	18	0.18	1.00	16	0.26	1.00
∆ TPA (cm^2^)	16	-0.01	0.98	17	0.13	1.00	15	0.20	1.00	13	-0.18	1.00
∆ TPV (mm^3^)	18	0.01	1.00	15	0.04	0.88	18	-0.01	0.96	16	-0.31	1.00
∆ VWV (mm^3^)	16	0.35	1.00	13	0.22	1.00	16	0.14	1.00	16	-0.22	1.00
∆ BMI (kg/m^2^)	19	-0.33	1.00	17	-0.54	0.30	18	-0.52	0.29	16	-0.06	1.00
∆ TC (mmol/L)	19	-0.18	1.00	17	0.15	1.00	18	0.28	1.00	16	-0.01	0.99
∆ HDL (mmol/L)	19	0.20	1.00	17	0.06	1.00	18	-0.08	1.00	16	0.03	1.00
∆ LDL (mmol/L	19	-0.30	1.00	17	0.06	1.00	18	0.24	1.00	16	-0.01	1.00
∆ TG (mmol/L)	19	-0.24	1.00	17	0.16	1.00	18	0.34	1.00	16	-0.01	1.00
∆ SBP (mmHg)	19	0.05	1.00	17	0.14	1.00	18	0.14	1.00	16	-0.07	1.00
∆ DBP (mmHg)	19	-0.33	1.00	17	-0.10	1.00	18	0.02	1.00	16	0.27	1.00

BMI = body mass index; TC = total cholesterol; HDL = high density lipoprotein; LDL = low density lipoprotein; TG = triglycerides; SBP = systolic blood pressure; DBP = diastolic blood pressure; IMT = intima-media thickness; TPA = total plaque area; TPV = total plaque volume; VWV = vessel wall volume; ∆, Delta = FU-BL.

**Table 5:** Relationship between change in ultrasound measurements and change in risk factors

**Change in risk factors**	**∆IMT (mm)**		**∆TPA (cm**^**2**^**)**		**∆TPV (mm**^**3**^**)**		**∆VWV (mm**^**3**^**)**	
	***r***	***p***	***r***	***p***	***r***	***p***	***r***	***p***
	**Group A**							
	*n* = 16		*n* = 18		*n* = 13		*n* = 12	
∆ BMI (kg/m^2^)	0.48	0.45	0.27	1.00	0.21	0.99	0.16	1.00
∆ TC (mmol/L)	-0.10	0.71	0.00	1.00	0.33	1.00	0.19	1.00
∆ HDL (mmol/L)	-0.23	1.00	0.13	1.00	0.46	0.81	0.07	1.00
∆ LDL (mmol/L	-0.18	1.00	0.14	1.00	0.21	1.00	0.17	1.00
∆ TG (mmol/L)	0.21	1.00	-0.03	1.00	0.06	0.85	0.28	1.00
∆ SBP (mmHg)	-0.19	1.00	-0.11	1.00	0.28	1.00	-0.04	0.91
∆ DBP (mmHg)	-0.39	0.83	0.06	1.00	-0.44	0.71	-0.12	1.00
	**Group B**							
	*n* = 19		*n* = 17		*n* = 18		*n* = 16	
∆ BMI (kg/m^2^)	-0.19	1.00	-0.26	1.00	0.02	1.00	-0.17	1.00
∆ TC (mmol/L)	0.23	1.00	-0.11	1.00	-0.21	1.00	-0.30	0.76
∆ HDL (mmol/L)	0.15	1.00	-0.08	1.00	0.02	0.95	-0.31	0.95
∆ LDL (mmol/L	0.25	1.00	-0.23	1.00	-0.30	1.00	-0.35	0.92
∆ TG (mmol/L)	0.14	1.00	0.30	1.00	-0.05	1.00	0.11	0.69
∆ SBP (mmHg)	0.02	0.93	-0.46	0.44	0.05	1.00	0.40	0.74
∆ DBP (mmHg)	0.55	0.11	0.06	0.82	-0.46	0.38	-0.44	0.60

BMI = body mass index; TC = total cholesterol; HDL = high density lipoprotein; LDL = low density lipoprotein; TG = triglycerides; SBP = systolic blood pressure; DBP = diastolic blood pressure; IMT = intima-media thickness; TPA = total plaque area; TPV = total plaque volume; VWV = vessel wall volume; ∆, Delta = FU-BL.

**Table 6:** Changes in US measurements for subjects with improved and unchanged TC/HDL

**Ultrasound measurements**	**Improved TC/HDL**		**Not improved TC/HDL**		***p***
	***n***	**Mean (SD)**	***n***	**Mean (SD)**	
∆IMT (mm)	23	0.001 (0.110)	12	0.065 (0.051)	0.28
∆TPA (cm^2^)	22	0 (0.25)	13	0 (0.33)	0.87
∆TPV (mm^3^)	20	1 (18)	11	-1(12)	1.00
∆VWV (mm^3^)	19	-9 (67)	9	37 (73)	1.00

BL = baseline; FU = follow-up; IMT = intima-media thickness; TPA = total plaque area; TPV = total plaque volume; VWV = vessel wall volume; TC = total cholesterol; HDL = high density lipoprotein; ∆, Delta = FU-BL.

## Discussion

Our goal was to investigate the potential changes in carotid atherosclerosis in subjects who had enrolled in a randomized controlled trial of six month CCR post-TIA [21]. We showed: 1) there were no significant changes in IMT, TPA, TPV and VWV in group A and B, and there were no significant differences in US measurements between groups A and B before or after therapy, 2) there were no significant correlations between baseline US measurements and the change in risk factors and, 3) there were no statistically significant differences in the change in US measurements between subjects with an improved and unchanged TC/HDL ratio.

First, we observed no significant changes in carotid ultrasound measurements of atherosclerosis in either groups A or B after 6 months of therapy. We note that a previous study [12] in a small group of 30 patients showed a significantly decreased TPV in high risk stroke subjects who administered 3-months intensive atorvastatin therapy as compared to those who received placebo. Another dietary intervention study also showed significant 2-year changes in 3DUS VWV a larger group of 140 subjects [14]. The fact that there were no significant changes in IMT, TPV or VWV here suggests that an extended period of CCR or perhaps a larger sample size is required to detect differences in carotid atherosclerosis measurements. Previous work that showed significant improvements in serum and other stroke risk factors [21] after 6 months CCR (although not controlled) also suggests that improvements in carotid atherosclerosis may require a longer time-frame or perhaps a larger sample size to detect.

As might be expected, we observed significant and modestly strong correlations between the 1D, 2D and 3D carotid ultrasound measurements. These results were similar to previous findings in different patient populations [24] and suggest that changes in IMT and TPA explain some of the variability in carotid TPV and VWW. This is not unexpected because TPV is in essence a 3D TPA measurement and VWV is essentially a 3D IMT + plaque measurement. It is also worth noting that there were significant linear correlations for male and female measurements when considered independently. These findings strengthen the conclusions derived from previous work [24] where a significant but weak correlation for TPV and VWV was observed in females.

Total cholesterol (TC) to high-density lipoprotein (HDL) cholesterol ratios are commonly used for evaluating the risk of stroke and can be used as predictors of peripheral arterial disease [27]. For the small number of subjects with improved TC:HDL ratio (n = 24), regardless of treatment group, there were no significant differences and this suggests that a larger sample size may be needed to show significant improvement in US measurements after 6 months therapy.

It is important to note the rationale for including carotid ultrasound imaging in this randomized controlled trial. While a previous study of the effects of CCR evaluated risk factors and plasma lipid biomarkers [21] and showed modest effects, carotid US measurements of atherosclerosis were included here to try to determine the direct effects of CCR on carotid artery media hypertrophy, intima thickening and atherosclerotic plaque. Carotid US measurements provide a robust, rapid and relatively inexpensive way to generate non-invasive measurements of the direct determinants of stroke and TIA risk.

We must acknowledge a number of study limitations. First, this was a small pilot study, involving only subjects who consented to US monitoring (39 in total) and of these, there were subjects with incomplete imaging data, mainly due to insufficient image quality to complete quantitative analysis for some of the US data. As previously described [21], subjects were also provided a choice between two exercise treatments. We think that this choice of treatment may have had an effect on outcomes, including results obtained from carotid ultrasound measurements. Among the different ultrasound measurements, we believe that VWV would be a strong candidate in monitoring atherosclerotic disease over time in individuals performing CCR. It offers a wide dynamic range and it provides information about the arterial wall thickness as well as any plaque that may be present in the area of interest around the bifurcation of the carotid artery. Future studies may be required with a larger number of subjects or perhaps a longer follow-up study to measure and follow stroke risk factors and 3DUS measurements. Further studies can be performed to evaluate how patients who have not yet experienced a stroke but are at high risk would respond to comprehensive cardiac rehabilitation. We would hypothesize an improved quality of life and risk factors, including those related to carotid atherosclerosis decreasing the likelihood of a stroke.

## Conclusions

We observed no significant difference in carotid US measurements of IMT, TPA, TPV and VWV within or between the two groups (A and B), after the 6-month evaluation period. A longer therapy period with an increased sample size may be required to show significant changes in US measurements in this patient population and after CCR.

## Abbreviations

1D: One-dimensional; 2D: Two-dimensional; 3D: Three-dimensional; 3DUS: Three-dimensional ultrasound; BF: Bifurcation; BL: Baseline; BMI: Body mass index; CCA: Common carotid artery; CCR: Comprehensive cardiac rehabilitation; DBP: Diastolic blood pressure; FU: Follow-up; HDL: High density lipoprotein; ICA: Internal carotid artery; IMT: Intima-media thickness; L: Left; LDL: Low density lipoprotein; LIB: Lumen-intima boundary; MAB: Media-adventitia boundary; R: Right; SBP: Systolic blood pressure; TC: Total cholesterol; TG: Triglycerides; TIA: Transient ischemic attack; TPA: Total plaque area; TPV: Total plaque volume; US: Ultrasound; VWV: Vessel wall volume.

## Competing interests

The authors declare that they have no competing interests.

## Authors’ contributions

TJL performed IMT, TPV and VWV measurements, presented and analyzed the data, and wrote the first draft of the manuscript. DB acquiring patient demographics and managed the patient information database, edited drafts of the manuscript and approved it. DP contributed to the analysis and revision of the manuscript. TH provided subject demographics and ultrasound images for the subjects involved in the study. NS, JDS, RDR, RC and MS generated the hypotheses and study concepts and reviewed and revised manuscript drafts. PLP reviewed the manuscript and generated hypotheses and study concepts for the RCT. GP, the corresponding author, supervised the data analysis and revised the manuscript. All authors approved of the manuscript.
